# The water kinetics of superabsorbent polymers during cement hydration and internal curing visualized and studied by NMR

**DOI:** 10.1038/s41598-017-10306-0

**Published:** 2017-08-25

**Authors:** D. Snoeck, L. Pel, N. De Belie

**Affiliations:** 1 Magnel Laboratory for Concrete Research, Department of Structural Engineering, Faculty of Engineering and Architecture, Ghent University, Tech Lane Ghent Science Park, Campus A, Technologiepark Zwijnaarde 904, B-9052 Gent, Belgium; 2 Transport in Permeable Media, Department of Applied Physics, Eindhoven University of Technology, Eindhoven, The Netherlands

## Abstract

SuperAbsorbent Polymers (SAPs) can be applied as an admixture in cementitious materials. As the polymers are able to swell, they will absorb part of the mixing water and can then release that water back towards the cementitious matrix for internal curing. This is interesting in terms of autogenous shrinkage mitigation as the internal relative humidity is maintained. The mechanism is theoretically described by the Powers and Brownyard model, but the kinetics and water release still remain subject of detailed investigation. This paper uses Nuclear Magnetic Resonance (NMR) to study the release of water from the superabsorbent polymers towards the cementitious matrix during cement hydration. The release of water by the SAPs is monitored as a function of time and degree of hydration. The internal humidity is also monitored in time by means of sensitive relative-humidity sensors.

## Introduction

SuperAbsorbent Polymers (SAPs) are a new promising admixture to be added to cementitious materials^1^. They are able to absorb several hundred times their own weight in fluids due to osmotic pressure and to retain it within their structure. They are used as controllable delivery systems with smart swelling and de-swelling properties^2^ which depend on the polymer properties and the characteristics of external solution such as ion concentrations, amongst others^3^. The water retaining property is also interesting for cementitious materials. SAPs can be used to increase the freeze-thaw resistance^4, 5^, to change the rheological behaviour^6^, for self-sealing^7–9^ and for self-healing^10–13^, amongst others.

They are also used to mitigate autogenous shrinkage in systems with a low water-to-binder ratio^14–18^. As, upon hydration of cement, the capillary water will be consumed followed by a reaction with the more strongly bound gel water, there will be self-desiccation leading to autogenous shrinkage. By internally or externally curing the material, this autogenous shrinkage can be prevented and the hydration can be maximized^19^. The SAPs can retain water, act as water-filled inclusions (macro pores) and release the water to reduce self-desiccation shrinkage during hardening. This internal curing will maintain the internal relative humidity. Due to the release of water by the SAPs, the SAPs decrease in size and afterwards a macro pore remains with the dry remnant of the SAP. When (external) water is available again, the SAP is able to swell to full extent.

The theory of Powers and Brownyard^20^ is used to describe the theoretical principle of internal curing. In a system with a high water-to-cement ratio (higher than 0.42^14, 20^), capillary water is available to obtain complete hydration. No autogenous shrinkage problems are observed. In a system with a low water-to-cement ratio (lower than 0.42), however, capillary water is completely used during hydration. The hydration continues with part of the stronger bound gel water, as cement hydration cannot proceed when a certain minimum porosity of the cement gel (26–28%) is reached^14^. When using SAPs for internal curing, no gel water is used. No autogenous shrinkage is found in such ideally internal-cured mixtures. Compared to a system with the same total water-to-cement ratio but without internal curing, the maximum degree of hydration is the same. The main difference is that in the SAP-entrained system, isolated macro pores are predominantly present compared to the finer capillary pore system present in the system without internal curing^21, 22^.

For internal curing of cementitious materials by SAPs, the SAP itself – i.e. type and size amongst others – plays an important role. If the SAP is releasing the water too fast (before final setting), the total water-to-cement ratio would increase. If the SAP is releasing the water too slow, it will keep its water and will not provide it to the cementitious matrix for internal curing. The SAPs thus need to release their water at the right time during hydration of the cementitious material. Furthermore, the SAPs need to be homogenously distributed and small enough to ensure a proper surface area available for internal curing. The ideal swollen size is in the range of 100–200 µm^14, 15^. A previous study^17^ has investigated a SAP within this range for internal curing, together with a less ideal bigger SAP still able to partially mitigate autogenous shrinkage. These two different sized SAPs are used in this paper.

The water release and kinetics from the SAPs during hydration are an important parameter to investigate. Mönnig^23^ visualised the densification of the cementitious matrix around a desorbing SAP particle when the material was hydrated. As the SAP particle releases its water, it will change the surrounding matrix and cause a shift towards finer pores^21, 22^. The SAP particle shrinks and an empty macro pore remains as shown by means of neutron tomography measurements^24^. The water release seems to coincide with the first drop in relative humidity^25^. Schröfl *et al*.^26^ showed the water release by means of neutron radiography measurements during hydration of the cementitious material.

Nuclear Magnetic Resonance (NMR) is a powerful technique to study the water amounts (^1^H) in a material. Even other elements can be studied, such as ^23^Na and ^35^Cl^27^. It can be applied to study the cement hydration^28, 29^ and fluid transport in porous materials^30^ as the hydrogen atom will have an interaction with the pore wall and as a result the signal will decay. The resulting relaxation rate is proportional to the pore structure^31^ and can be quantified as well. Moisture profiles of porous materials during heating could be studied and conclusions could be drawn on how the water escaped the sample as a function of time^32, 33^. The profiles can be used to study drying or saturation of a mortar sample^34, 35^, the water release from a cement paste to a porous material^36^ or for autogenous healing^37^. Only a few studies utilizing NMR relaxometry for water release by SAPs towards the cementitious matrix have been done. Friedemann *et al*.^38^ showed that water is available during post-internal curing of cement pastes. Nestle *et al*.^39^ studied the water balance in a cement paste with SAPs. The release of water from the SAPs towards the cementitious matrix started by the time of the acceleration period of cement hydration and lasted for two days. Still, the kinetics of water release of the SAPs towards the cementitious matrix need to be investigated in detail to fully understand the principle of internal curing and the effect the SAPs have on and during internal curing. By using the non-destructive NMR test, the differences in water states and pore size distribution can be investigated and compared in systems with and without SAPs.

In this paper, the water release by the SAPs and the change in water states are monitored by means of NMR tests as a function of time and degree of hydration. The results are compared to the theoretical model of Powers and Brownyard and supported by measuring the internal relative humidity.

## Materials and Methods

In this section, the different studied mixtures are explained (2.2). These mixtures are chosen to study the release of entrained water by the SAPs for internal curing. For that, the swelling capacity of the SAPs and their swelling times need to be assessed first (2.1). Next, the NMR principle and apparatus together with the performed calculations to investigate the release kinetics from the SAPs to the cementitious matrix are explained (2.3). In the following part the hydration and setting properties are discussed in order to compare the obtained results with the Powers and Brownyard model (2.4). In the end, information about the internal relative humidity measurements is provided which will serve as a basis of reference of the mitigation of self-desiccation and the link to the NMR results (2.5).

### SAP absorption characteristics and added SAP amounts

To determine the amount of SAPs needed to absorb the amount of entrained water, the swelling capacities need to be known. This swelling capacity can be studied in different ways and with different methods. A recent review^40^ combines all these tests and points towards two main tests which can be conducted: the tea-bag test^41^ and the filtration test^13^. The latter was conducted in this research. The test fluids were demineralized water (pH = 6.5) and a cement filtrate solution. For the cement filtrate solution 100 g of cement was mixed in 1 l of demineralized water and subsequently filtered after 6 h. The filtered solution (pH = 12.8) was used for testing. The cement used was CEM I 52.5 N and the chemical composition can be found in Table 1.

**Table 1:** Chemical and mineral composition of CEM I 52.5 N and specific surface (Blaine fineness).

	*CEM I 52.5* *N mass-%*
CaO	63.12
SiO_2_	18.73
Al_2_O_3_	4.94
Fe_2_O_3_	3.99
SO_3_	3.07
MgO	1.02
K_2_O	0.77
Na_2_O	0.41
Cl^−^	—
S^2−^	—
Mn	—
C_3_S	67.0
C_2_S	3.2
C_3_A	6.3
C_4_AF	12.1
Specific surface	390 m²/kg

For the filtration method^13^ a known weight of SAP (m_1_) is added to an excess of testing fluid (m_2_). After 24 h the whole is filtered and the mass of filtered fluid is recorded (m_3_). During the test, the filter paper was pre-saturated and the testing setup was covered to minimize possible carbonation and evaporation. The absorbed mass of testing fluid per gram of dry SAP equals: (m_2_-m_3_)/m_1_. The test was performed in tenfold (n = 10). The results are shown in Table 2. Two SAPs (obtained from BASF) were investigated. These were SAP A, a cross-linked copolymer of acrylamide and sodium acrylate (particle size 100.0 ± 21.5 µm (n = 100)), and SAP B, a cross-linked potassium salt polyacrylate (particle size 476.6 ± 52.9 μm (n = 100)). Both SAPs are bulk-polymerized and consist of irregular crushed particles.

**Table 2:** The absorption capacity of SAP (n = 3) in demineralized water and cement slurry [g/g SAP], absorption capacity of SAP during mixing of a cement paste [g/g SAP], and swelling time until full saturation of particles is achieved [s] with respective standard deviations.

*Method*	*SAP A*	*SAP B*
Δm/m de-ionised water (pH = 6.5)	305.0 ± 3.7 g/g SAP	283.2 ± 2.4 g/g SAP
Δm/m cement slurry (pH = 12.8)	61.0 ± 1.0 g/g SAP	58.4 ± 1.7 g/g SAP
Δm/m during cement paste mixing	23 g/g SAP	11 g/g SAP
Swelling time	10 ± 2 s	60 ± 5 s

To determine the swelling time, the vortex method was applied based on the one found in literature^42^. For this test, 100 g of demineralized water was added to a beaker. Then, a vortex was made using a magnetic stirrer (20 mm length at 400 rpm). From the already obtained absorption capacity, the specific amount of SAPs to absorb 100 g of demineralized water was added to the beaker. The time was recorded until the vortex disappeared (n = 10). This time served as a reference of the time of swelling.

The amount of mixing water absorbed was calculated from the comparison of the flow values of the different mixtures, following the Standard EN 12350-5. This influence on the flow values reflects the absorption of the SAPs in the mortar mixture^22, 43^. These values need to be interpreted with precaution as the SAPs may absorb more water or release water prior to setting. The tests were conducted after 5 min of water addition to the cement. This corresponds to a time when the total swelling (in the order of seconds see Table 2) was already reached. Additionally, microscopic analysis was conducted to verify whether the formed macro pores had the expected size^22, 44, 45^. When assuming spherical SAPs, the swollen size can be calculated starting from the initial dry size and the amount of absorbed entrained water and compared to the found macro pores. In that way, the swelling capacity in the mortar could be determined.

### Studied mixture compositions

Two reference cement pastes with a water-to-binder ratio (W/B) of 0.3 (R0.30) and 0.354 (R0.354), respectively, were made. Superplasticizer was added in an amount of 0.5 and 0.3 m% (mass-% of cement weight) to ensure practical workability for the R0.30 and R0.354 mixture. The superplasticizer used was a polycarboxylate type (Glenium 51, conc. 35%, BASF).

The R0.30 mixture has a low water-to-cement ratio and is prone to autogenous shrinkage. Based on the model of Powers and Brownyard, the amount of needed entrained water in the SAPs for internal curing can be theoretically calculated and amounts to an additional water-to-cement ratio of 0.054^14^. In that way, the total water-to-cement ratio (w/c)_tot_ equals 0.354, but with the entrained water-to-cement ratio (w/c)_e_ it leads to an effective water-to-cement ratio (w/c)_eff_ of 0.30.

Two different mixtures containing different SAPs were investigated. These were SAP A (cement paste Ae) and SAP B (cement paste Be). All SAPs were stored in dry and sealed conditions prior to testing or mixing in the cement paste mixture. The SAP particles were added on top to the cement and were first dry mixed to ensure a homogenous dispersion in the cement. After this dry mixing, the total water was added together with superplasticizer which was dissolved in the water prior to addition. The amount of superplasticizer in the SAP mixtures was the same as for the R0.30 mixture, to minimize the influence on the setting properties of the respective mixtures^46^. The amount of SAP to be added, after conducting the above-mentioned tests based on the mixing water absorption in section 2.1 was 0.22 m% (mass-% of binder weight) SAP A (Ae) and 0.45 m% SAP B (Be), respectively. There was no loss in workability observed.

### Nuclear Magnetic Resonance (NMR) setup

**Figure 1:**
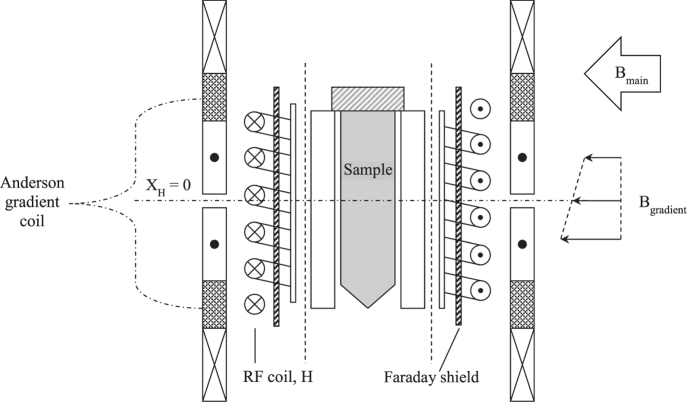
The NMR probe head for measuring the hydration. Sealed sample containers of ∅ 27 mm and 100 mm length filled with the cement paste are used. A Faraday shield has been added to make the NMR measurements quantitative. Using an Anderson gradient coil a gradient of 0.3 T/m is applied giving a resolution in the order of 1 mm.

The sealed sample containers (cylinder ∅ 27 mm × 100 mm high) filled with the cement pastes were put in the NMR setup as shown in Fig. 1. An external magnetic field B_0_ was applied of 0.8 T corresponding to a frequency of 34 MHz. This field was provided by a water-cooled iron-cored electromagnet with poles 50 mm apart from each other. A coil was placed around the sample for creating and receiving the radiofrequency fields during an NMR measurement. A Faraday shield was added between the coil and the sample to suppress the effect of the changes of the dielectric permittivity by variations of the moisture content. In that way, the NMR measurements are made quantitative. All NMR measurements were performed at room temperature (23 ± 1 °C).

NMR is a magnetic resonance technique in which the magnetic moments of the nuclei are manipulated. The frequency of the condition is given by the Larmor frequency f_1_:

1 $${f}_{1}=\frac{\gamma }{2\cdot \pi }\cdot {B}_{0}$$

Where B_0_ is the externally applied static magnetic field and γ is the gyromagnetic ratio. The gyromagnetic ratio depends on the type of nucleus and for ^1^H γ/2π is equal to 42.58 MHz·T^−1^. Hence NMR can be made sensitive to only hydrogen. The magnetic field gradient was set at 0.3 T/m offering a one-dimensional resolution in the order of magnitude of 1.0 mm. In a so-called pulsed NMR experiment the magnetic moments of the hydrogen nuclei are manipulated by Radio Frequency (RF) pulses at the resonance frequency. The amplitude of the so-called Hahn spin-echo signal is proportional to the hydrogen density. Moreover the spin-echo signal also provides information about the rate at which the magnetic excitation of the spins decays.

For the moisture signal, a Hahn-spin echo signal is obtained with signal intensity S equal to:

2 $$S=k\cdot \rho \cdot [1-exp(-\frac{{T}_{R}}{{T}_{1}})]\cdot exp(-\frac{{T}_{E}}{{T}_{2}})$$

where k is a proportional constant, ρ is the density of the nucleus, T_1_ the spin-lattice relaxation and T_2_ the spin-spin relaxation. The T_1_ reflects the relaxation due to interactions of spins with the surrounding matrix, whereas T_2_ reflects spin-spin interactions. Both the T_1_ and the T_2_ can give information about the distribution of the pore sizes in a porous material^34^. The repetition time T_R_ and the echo time T_E_ are also important parameters. The spin-echo signal S is recorded after a time T_E_ equal to 180 µs. The relaxation time T_2_ of water in a porous material can be as short as 500 µs^32^ and S needs to be recorded before it has decayed. T_R_ is the time in between two successive spin echo experiments and typically T_R_ > 4·T_1_ is chosen.

The Hahn spin-echo was always determined prior of testing to decide for the parameters to be used. The following parameters were used: 29.422 MHz center frequency, 30 µs pulse time, 180 µs echo time and 64 averages. In this study T_2_ was used to determine the pore-size development during hydration which was measured using Carr-Purcell-Meiboom-Gill sequences (CPMG). For CPMG following additional parameters were used: 1000 ms repetition time and 128 echoes for R0.30 and R0.354. For Ae and Be these values were 2000 ms and 2048 echoes, respectively. The resulting measured relaxation represents a complex summation of decaying signals. The data analysis of such signal involved a Laplace inversion to obtain a distribution of relaxation times or diffusion constants. In this paper, Fast Laplace Inversion (FLI)^47^ was used to obtain the relaxation times T_2_ as a spectrum.

All signals were normalized to the first signal to receive the percentage. The weighed percentage of each peak was then multiplied with the normalized signal to receive the relative ratio of each studied peak as a function of time. This was also studied as a function of the degree of hydration (see next section) and plotted on the theoretical model of Powers and Brownyard. The used densities for the calculation from the mass to the volume percentage of the Powers and Brownyard model were 3140 kg·m^−3^ cement, 1000 kg·m^−3^ water, 1100 kg·m^−3^ superplasticizer and 1400 kg·m^−3^ SAP.

For the SAP-mixtures, a comparison was made between the signal of the SAP entrained water and the free water to see when and how the water is released by the SAPs for internal curing.

Furthermore, T_2_ is proportional to the pore size distribution due to the following relation^30, 31, 34^:

3 $${T}_{2}=\frac{V}{S\cdot {\rho }_{2}}$$

where V is the volume, S is the surface and ρ_2_ surface relaxivity. Assuming spherical pores with a diameter d, the volume-to-surface ratio is equal to d/6. Müller *et al*.^28^ used planar pores of area A where S = 2·A, V = A·d and the volume to surface ratio is equal to d/2. They used a surface relaxivity of 3.73·10^−3^ nm·µs^−1^ for a white Portland cement. The latter two are also used in this research. From the obtained T_2_ spectra the peaks are located and multiplied by the volume-to-surface ratio and divided by the surface relaxivity.

### Setting and hydration modelling

The Vicat needle test following the Standard ASTM C191 – 08 (Method A: Reference Test Method using the manually operated Vicat apparatus) was used to determine the final setting of the mixtures. By periodically penetrating (n = 3) the thin cement paste with the 1-mm Vicat needle, the time between initial contact of cement and water and the time at which the needle did not leave a circular impression in the paste surface, was recorded and used as the time of final setting. This time of final setting is often used as the starting point of conduction autogenous shrinkage measurements^17^ and is used to give referential information of the time of water release by the SAPs.

Via the chemical composition (as found in Table 1) the mineral composition was calculated using the Bogue equations. The model of Parrot and Killoh^48^ was used to determine the degree of hydration. The model uses the water-to-cement ratio and the mineral composition of the cement. In that way, the degree of hydration was obtained. This degree of hydration was then linked to the age of the sample during testing and this was used to link it to the intensity of the NMR signal. In that way, the model of Powers and Brownyard could be verified based on the obtained NMR data starting from the initial known amounts of water and the respective densities.

The maximum degree of hydration α_max_ was also theoretically calculated from Powers and Brownyard’s model using equations 4–5 ^20, 49^. When there is internal curing, equations 5–6 is used with the effective water-to-cement ratio or equations 4–5 with the total water-to-cement ratio.

4 $${\alpha }_{max}=\frac{p}{1.32\cdot (1-p)}$$

5 $$p=\frac{(w/c)}{{\rho }_{c}+(w/c)}$$

6 $${\alpha }_{max}=\frac{p}{1.12\cdot (1-p)}$$

where ρ_c_ is the specific volume of cement. For R0.30 α_max_ is 0.72 and for R0.354, Ae and Be α_max_ is equal to 0.85. The obtained values were used to visualize the theoretical model of Powers and Brownyard.

### Internal relative humidity measurement

To see whether the SAPs were able to mitigate self-desiccation (and thus autogenous shrinkage), the internal relative humidity was monitored. Information on the effects of autogenous shrinkage mitigation of the two SAPs can be found in Snoeck *et al*.^17^.

The internal relative humidity was monitored using sensitive relative humidity sensors. These humidity and temperature probes were obtained from Vaisala (HMP110). They have a measurement range of 0% to 100%RH and −40 °C to + 80 °C. The probe has a Vaisala Humicap 180 R sensor with an accuracy of ± 1.5%RH. The temperature sensor is a Pt1000 RTD Class F0.1 IEC 60751 with accuracy of ± 0.2 °C. Together with the probes, a protective lid system was used to protect the probes and to maintain the internal conditions of the sample near the probe.

**Figure 2:**
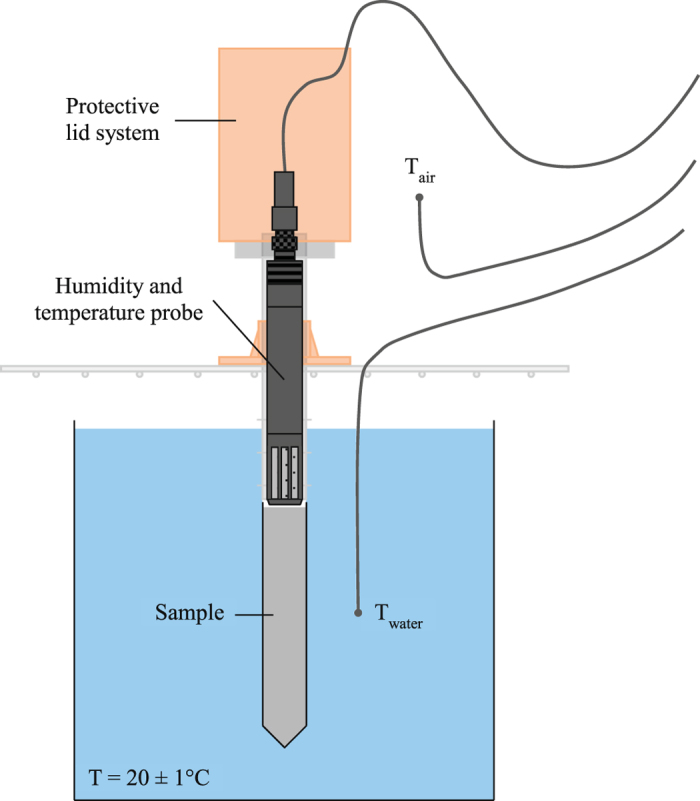
Used relative humidity and temperature probe to monitor the internal environment of a sample container (cylinder ∅ 14 mm × 85 mm high) filled with the cement paste with protective assembly and used thermocouples to record the ambient temperature in the water bath and the surrounding climate chamber.

The cement paste was poured in a small cylinder (∅ 14 mm × 85 mm high) and the probe was immediately placed hovering above the paste (Fig. 2). The protective assembled lids were used and the connection between the cylinder and the assembly, and the top was sealed with parafilm to exclude moisture escaping from the setup. In that way, a sealed environment was obtained and the relative humidity and temperature could be monitored as a function of time. The complete setup was placed on a metal grid in such a way that it was vertical for the complete measuring time.

To cancel the effect of the hydration heat – i.e. a small increase in temperature such as the heat of hydration may shift the RH measurements to false readings – the setup was submerged in a water bath placed at constant 20 ± 1 °C.

### Data availability statement

The datasets generated during and/or analysed during the current study are available from the corresponding author on reasonable request.

## Results and Discussion

In this section, the relaxation distribution and the percentage of signal intensities are discussed first (3.1) to provide information on the kinetics of SAP entrained water release for internal curing. Next, the results are linked to the Powers and Brownyard model (3.2) and the calculated pore size distributions compared to existing models (3.3). In the end, the effectiveness of an SAP for internal curing is discussed and supported by means of additional internal relative humidity measurements (3.4).

### Relaxation distribution and percentage of signal intensity

**Figure 3:**
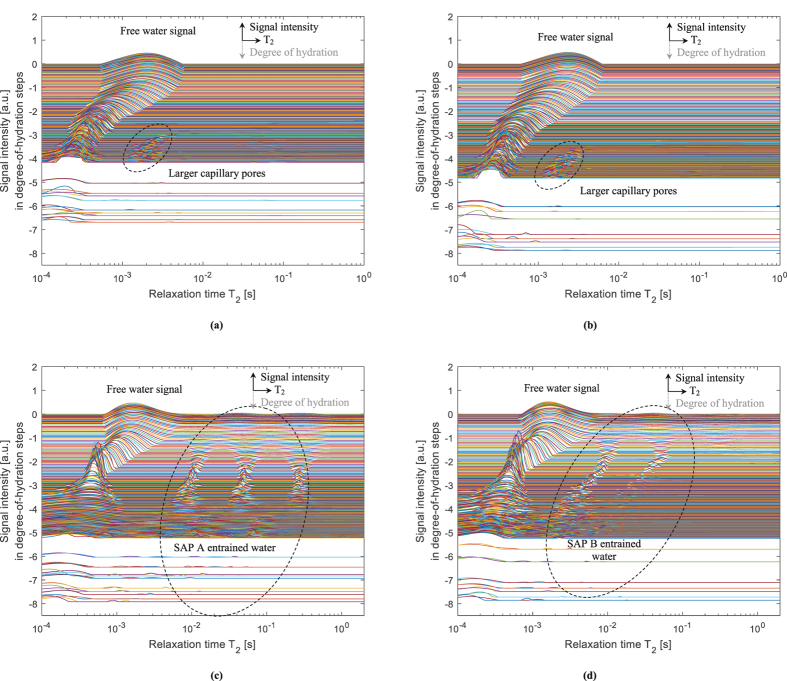
Signal intensity [a.u.] as a function of the relaxation time T_2_ [s] and in steps of the degree of hydration from top to bottom showing different allocated peaks in the R0.30 **(a)**, R0.354 **(b)**, Ae **(c)** and Be **(d)** mixtures. The time step in between curves is approximately 10 min up to an age of 2-3 days, followed by a measurement every two days up to a week, every week up to a month and every two weeks up to two months.

When a cement paste is hardening, the water will be consumed and products will be formed causing the material to become denser. This is seen in Fig. 3 showing the T_2_ relaxation distribution profiles for all studied specimens. They are shifted downwards as a function of the degree of hydration to visually show the change in time and to compare the obtained results. The distinct peak in all systems is attributed to free water. In the reference samples, we can clearly see a shift in relaxation time from the 10^−3^ s to 10^−4^ s range as a function of the degree of hydration. Furthermore, a smaller peak is formed at higher degree of hydration in the 10^−3^ s range. This latter peak may be attributed to some larger capillary pores which are visible after hardening of the cement paste due to the decrease in intensity of the other predominant peak.

In the SAP systems, additional peaks are allocated to SAP-entrained water in the 10^−2^ s to 10^0^ s range. This water is stored in the SAP particles and can be released for internal curing. In that way, as the T_2_ is different, the water kinetics from the SAPs towards the cementitious matrix could be studied in detail as a function of time and as function of the degree of hydration. For the SAP A mixture (Ae), it is clear that water is still present in the SAP inclusions at later ages (after final setting); i.e. at higher values of the degree of hydration in the lower positioned curves. For SAP B (Be), this is not entirely the case and the signal for entrained water drops as a function of time and thus as a function of the degree of hydration. From this relaxation time spectrum, it is already clear that the SAP A system seems to be promising for internal curing, as for the SAP B systems, water is consumed quickly.

**Figure 4:**
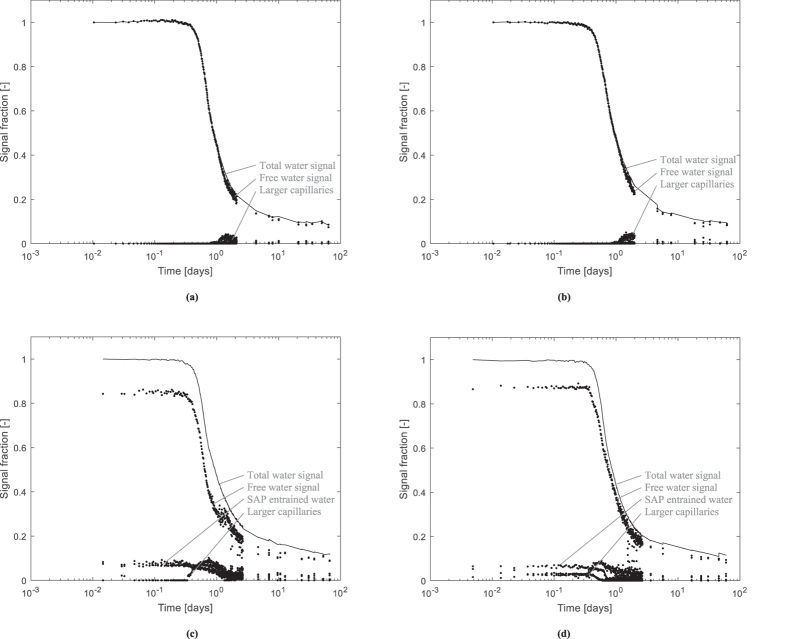
Signal fraction [−] of the free water peak and the SAP peaks as a function of the logarithmic time [days] showing the cement reaction and, where appropriate, internal curing in the R0.30 **(a)**, R0.354 **(b)**, Ae **(c)** and Be **(d)** mixtures.

The signals were then transformed towards percentages of the initial signal intensity. In that way, a gradational graph was obtained showing the height of the different peaks relative to the initial signal intensity. These results are shown in Fig. 4. The largest peak from the free water has the largest portion of the total signal fraction. In time, this water is used and consumed for cement hydration causing the total signal intensity to drop in time. Initial setting occurred at approximately 8 h of age and final setting was at approximately 11 h of age as determined with the Vicat needle test. The time of initial setting corresponds to the drop in NMR intensity signal. From this point onwards, the free water was used more visibly in NMR in all studied samples.

After final setting, the SAP-entrained water was visibly consumed, i.e. for internal curing. The signal intensities for SAP A decreased more steadily compared to the signals for the SAP B mixture. Here, water was more quickly consumed and used in the overall system. As SAP A is ideal to mitigate autogenous shrinkage and SAP B only mitigates to a minor degree^17^, already a glimpse of the mechanisms governing internal curing can be seen by the decrease in T_2_ spectrum peaks.

By comparing the relative signal intensities of the entrained water in the SAPs and the free water and dividing both by the total intensity, values of 84.3% and 85.2% free water and 15.7% and 14.8% entrained water are found for the SAP A and SAP B system, respectively. This is comparable to the theoretical values when dividing the amount of entrained water by the total amount of mixing water; 5.4/35.4 = 15.3%. The effective water-to-cement ratio is hereby 0.30, the entrained water-to-cement ratio 0.054 and the total water-to-cement ratio 0.354. This means that the amount of free water not present in the SAP is 30/35.4 = 84.7%. There is a good correlation between these theoretical values and the values obtained with NMR. The amount of SAP was determined in such a way that the absorption as determined by the flow values, autogenous shrinkage mitigation and by means of microscopic analysis was the correct one^17^.

As the cementitious matrix is formed at the time of final setting, the respective macro pores were found by image analysis on polished cross sections of hardened cement paste. The size was the one (257 µm for SAP A and 981 µm for SAP B) which could be expected when comparing the swelling capacity (mixing water in Table 2) and the initial dry size of the SAP particles. However, from this test the real behaviour of the SAP, i.e. faster release of entrained water from SAP B compared to SAP A is not detectable. This caused the SAP B polymer to realize less autogenous shrinkage mitigation, as reported in literature^17^. This shows the strength of using NMR to determine the real water kinetics from the SAPs for internal curing.

### Link with Powers and Brownyard model

**Figure 5:**
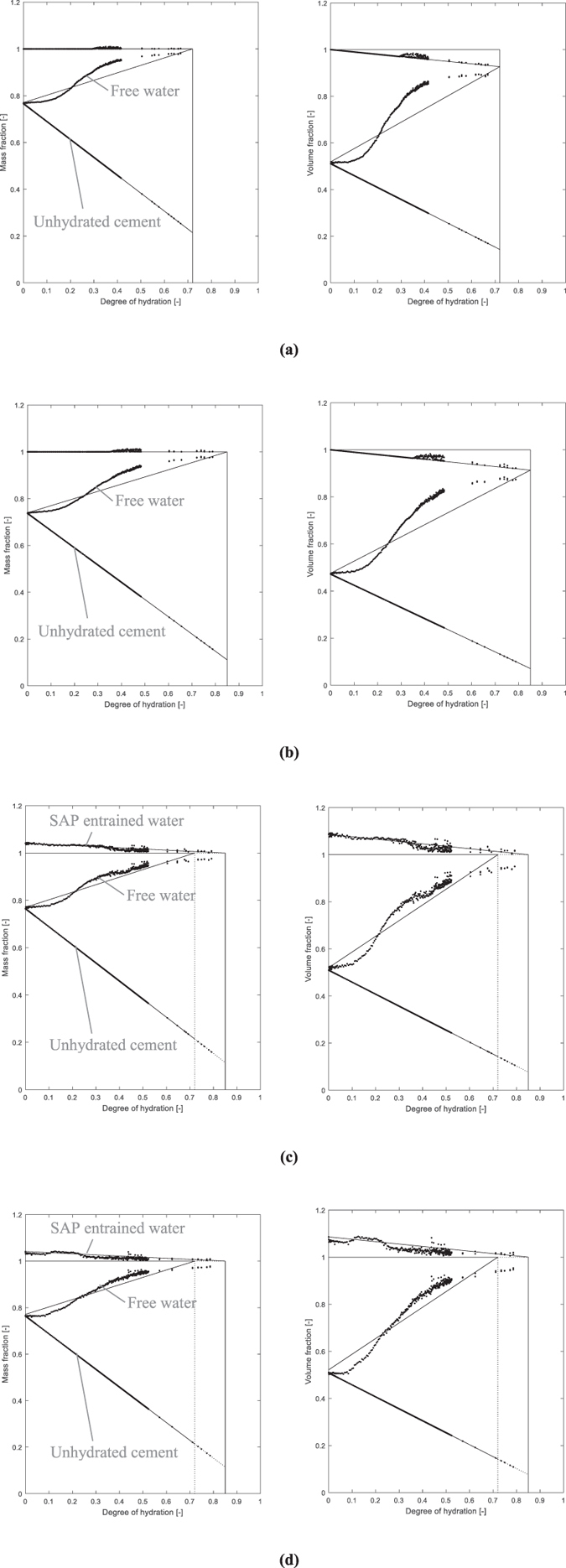
Mass (left) and volume (right) fraction [−] of the free water and the entrained water in the SAPs as a function of the degree of hydration [−] showing the cement reaction and where appropriate internal curing in the R0.30 **(a)**, R0.354 **(b)**, Ae **(c)** and Be **(d)** mixtures.

The found signal intensity results were plotted on the theoretical lines described in the model of Powers and Brownyard^20^ as a function of the degree of hydration and can be found in Fig. 5 both as mass and volume fraction. The consumption of free water for cement hydration can again be clearly seen.

The obtained NMR results correspond nicely to the lines proposed by Powers and Brownyard. Recently, results found in literature^28, 29^ suggest that the free water line is not a straight line as stated in the model. A more concave downward curve is expected as found by means of NMR on cement pastes with white cement. These results correspond with the plotted curve in this research from a degree of hydration of 0.2 upwards. A more steady change in behaviour prior to 0.2 degree of hydration is found in this research. On the top horizontal curve, some noise is found due to small observed peaks in the spectrum as found in Fig. 3.

From the results of the SAP mixtures plotted on the theoretical Powers and Brownyard’s lines, the entrained water stored in SAP A completely follows the theoretical line. For SAP B, this is also the case, but from a degree of hydration of 0.3 onwards, the curve slightly moves downwards. Part of the entrained water is thus prematurely released towards the cementitious matrix for internal curing. It is also possible that the detachment of the swollen SAP from the faces of the macro pore can cause a decrease in signal, but as the water spin-spin mechanism is governing in this measurement, this only has a minor influence. The release of entrained water from SAP B was also clearly visible as disappearing peaks in the T_2_ spectrum found in Fig. 3d.

### Pore size distribution and link to existing models

**Figure 6:**
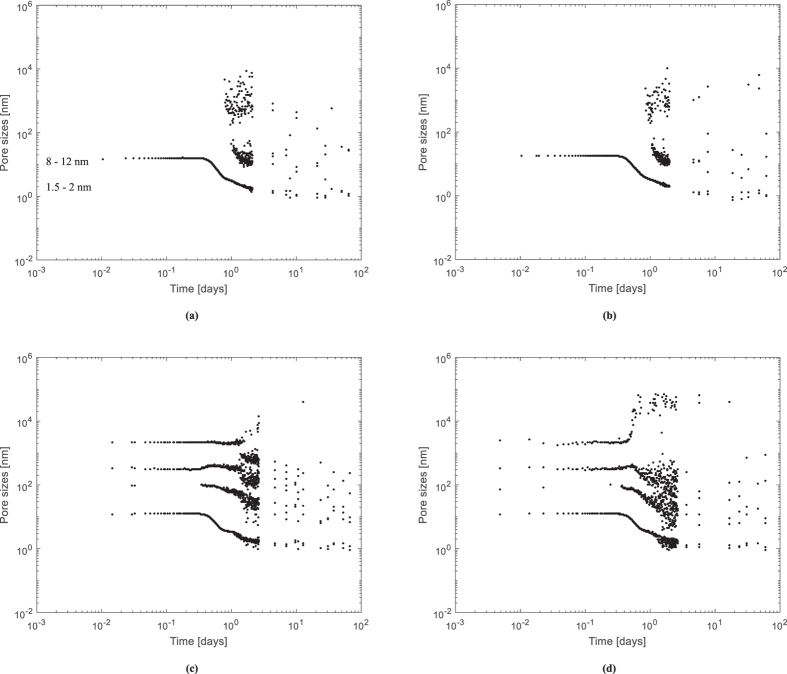
Pore size distribution [nm] obtained from the relaxation times T_2_ as a function of the logarithm of time [days] for the R0.30 **(a)**, R0.354 **(b)**, Ae **(c)** and Be **(d)** mixtures.

The water content in the pores is found in the T_2_ spectrum intensities and can be studied with time during hydration of the cement paste. T_2_ is proportional to the pore size distribution^30, 31, 34^ by multiplying the index of the found peak with the surface relaxivity and assuming planar pores. The pore size distribution results are found in Fig. 6. In time, a decrease in pore sizes is observed. This represents the densification of the cement paste due to hydration.

For the R0.30 mixture the main pore sizes found are in the range of 1.5–2 nm followed by an amount of pores with size of 8–12 nm, representing gel pores. Furthermore, some bigger capillary pores (10 to 1000 nm) are found as well. The microstructure of mortar studied by NMR in literature shows small (<10 nm) gel pores and bigger (10 to 1000 nm) capillary pores^34^. The T_2_ values are also comparable to the values found by Müller *et al*.^28^. The latter addressed the found pores of 2.5 nm to gel pores, 8 nm to interhydrate pores. This is also the range of pores we found. The size of the pore containing the free water stabilizes at around 8–12 nm.

The model of Feldman and Sereda^50^ shows mainly interlayer water and physically adsorbed water but no larger intrinsic reservoirs in C-S-H. They describe C-S-H as quasi-continuous layers with nanometer sized irregularities. The found pore size distribution also fits the Jennings Colloidal Model (CM-II) for C-S-H^51, 52^. He proposes three different pore size categories. The first are the intraglobular pores (<1 nm). The second are the small gel pores that exist outside the globules (<3 nm) and the third are the larger gel pores (3–12 nm). It appears that the first and the second category are found in the spectral array of T_2_ around the 1.5–2 nm scatter as obtained by NMR. The third category is seen as the pores with sizes in the range of 8 nm to 12 nm.

Clear peaks in T_2_ were assigned to the entrained water in Fig. 3, even though the sizes are not corresponding with the macro pore size of the swollen SAP particle (around 257 µm in the swollen state within the cementitious material) if a calculation is made starting from the obtained T_2_ (<1000 nm) and as shown in Fig. 6c,d. A reason stated in literature is the intrusion of solutes from the cement pore water in the SAP which might lead to enhanced relaxation due to paramagnetism and/or the formation of precipitates in the SAP^38^. With a Hahn spin-echo experiment of pure SAP in cement filtrate solution, we could not observe such T_2_ relaxation times. Furthermore, as the signal does not decrease substantially in time, these reasons could not occur. The alkaline environment in the SAP is continuously changing^41^ and no visible precipitates are found when looking at SAPs in macro pores in polished sections. Another reason for the observed SAP T_2_ peaks could be that water molecules in the SAP can have diffusive exchange with water molecules at the interface with the cementitious matrix^39^. As water diffusion is almost not impeded in a SAP particle, as studied by means of a single Hahn spin-echo test, this is the main explanation for the found peaks in the T_2_ spectrum. As the SAP particles are irregular in size, a scatter in this diffusive exchange is found.

### Effectiveness of an SAP for internal curing

**Figure 7:**
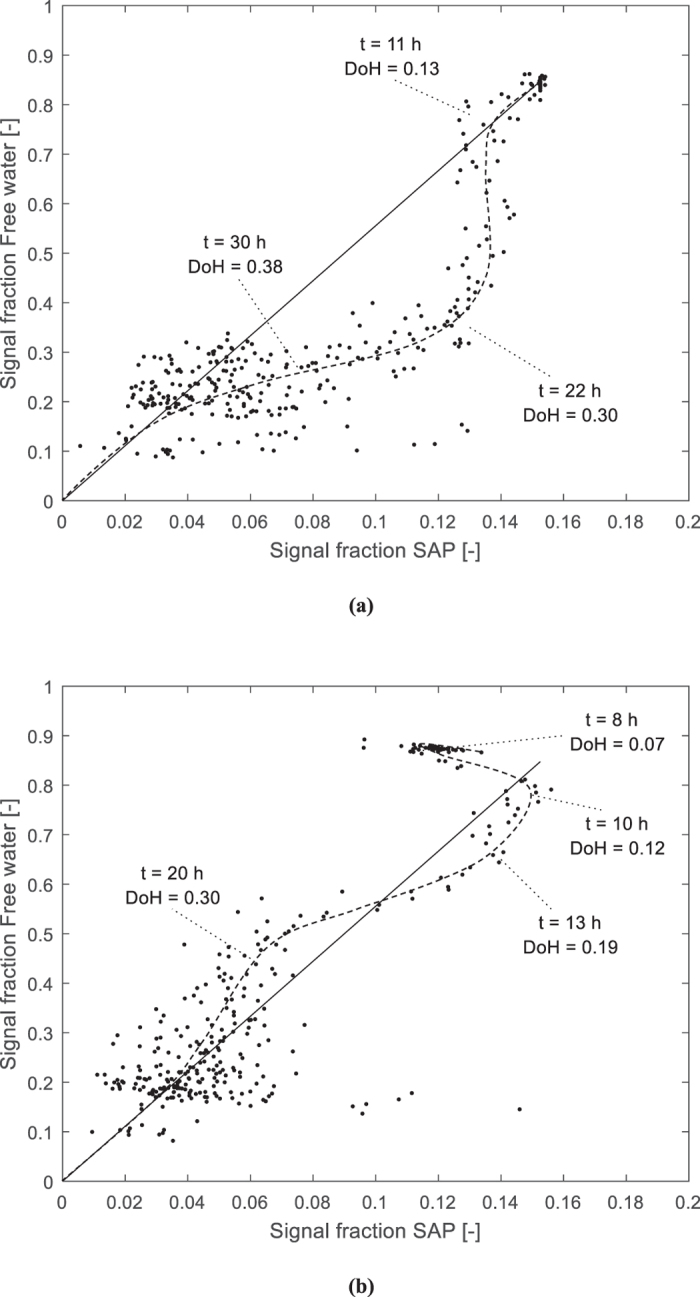
Comparison of the signal fraction of entrained water in the SAPs [−] to the signal fraction of free water [−] for the Ae **(a)** and Be **(b)** mixtures showing a different kind of behavior towards internal curing.

To study the kinetics of the SAPs for internal curing, the relative signal intensities for the entrained water and the free water could be compared. The results are shown in Fig. 7. In this figure, the overall theoretical relation is also depicted. This is 30/35.4 divided by 5.4/35.4 or 30/5.4 = 5.56. This is the theoretical ratio found by using the Powers and Brownyard’s model. However, as stated above in section 3.2, the line for the free water is not straight. The ratio, however, was used for a general way of comparison of the different results found in this research.

When looking at the results found for SAP A mixtures (Fig. 7a), the SAP A seems to release little amount of entrained water in the beginning (approximately at 11 h and 0.13 degree of hydration), after final set. Then the free water is consumed and at some point (approximately at 22 h and 0.30 degree of hydration), the entrained-water signal in the SAPs is decreasing again (till approximately 30 h and 0.38 degree of hydration), and the overall ratio is moving towards the theoretical one. For SAP B (Fig. 7b), this is not the case. The SAPs seem to release their stored water prematurely before initial setting (approximately at 8 h and 0.07 degree of hydration), to re-absorb part of the free water before final setting (approximately at 10 h and 0.12 degree of hydration) and again release it more quickly before the more pronounced decrease in free water (approximately from 13 h till 20 h and 0.19 till 0.30 degree of hydration). In the end, the ratio is again the same, and comparable to the SAP A mixture. However, as part of the entrained water is already released, there will be less mitigation of autogenous shrinkage or maintaining of the internal relative humidity.

**Figure 8:**
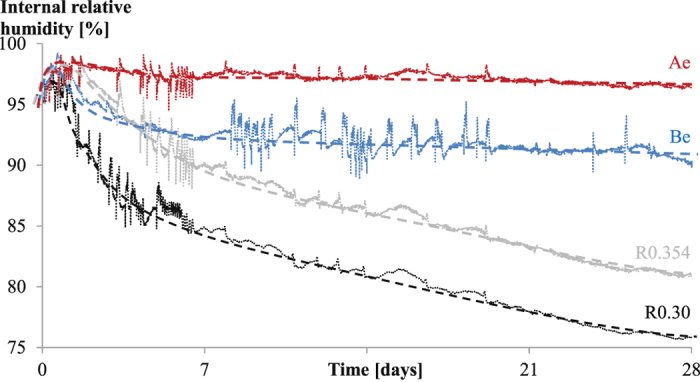
Internal relative humidity [%] as a function of time [days] showing the positive effect of internal curing for mitigation of self-desiccation for all studied mixtures.

When studying the internal relative humidity in Fig. 8, a different trend for each series is observed. After setting, the RH in reference samples starts to drop. This drop is more pronounced for a mixture with a lower water-to-cement ratio. The relative humidity keeps decreasing as a function of time and the rate for R0.30 and R0.354 after seven days is the same. For the SAP mixtures, the trend is different. For Be there is a partial drop in RH followed by a constant RH. This means internal curing is taking place, but not to its full extent. This could be due to the large particle size of SAP B and thus the lower surface area available for internal curing as compared to the smaller SAP A. Important is the inter-particle spacing as the water transport takes place within 2 mm from the internal reservoir containing the internal curing water^53^. The ideal size of the SAP for internal curing is within the range of 100 µm to 200 µm^14^, which is more the case for SAP A (257 µm in swollen state within the cementitious matrix) while it is much higher for SAP B (981 µm in the swollen state within the cementitious matrix). The smaller SAPs are better distributed throughout the cementitious matrix and are effectively causing internal curing (Ae). The internal RH does not decrease too much after 28 days. The measured RH at 28 days is 96%, 90%, 80% and 76% for Ae, Be, R0.354 and R0.30, respectively. These results are comparable to the curves found by means of neutron radiography^54^. The resolution used in the latter research was, however, too low to really study the water release by the SAPs towards the cementitious matrix.

The results are also substantiating the results found with NMR. As confirmed by the results plotted on the model of Powers and Brownyard (Fig. 5), SAP A is ideally maintaining the internal RH and thus mitigating autogenous shrinkage. For SAP B, it partially maintains the internal RH and thus mitigates autogenous shrinkage partially. The quick drop in RH for the SAP B mixtures seems to correspond to the observed quick drop in signal intensity for the entrained water stored in the SAP B particles (Fig. 3).

These results correspond to autogenous shrinkage measurements on the same mixtures within corrugated tubes^17^. In the latter results, the R0.30 mixture and R0.354 mixture showed autogenous shrinkage. Most shrinkage was found in the R0.30 system as the cementitious matrix is denser and there thus is an increase in self-desiccation due to the higher hydrostatic tension forces in the capillaries. For Ae the autogenous shrinkage was completely mitigated. In the paper^17^ it was opinioned that SAP B was releasing its water too slow. In this paper, it is shown that it releases its water too fast, from final setting onwards.

## Conclusions

Based on the findings of the research on internal curing by SAPs as studied by means of NMR following conclusions can be drawn:

- The free water and the entrained water by the SAPs could clearly be distinguished in the T_2_ relaxation spectra. The SAP signals relate to the exchange of their entrained water molecules with water molecules at the interface with the cementitious matrix. The signal for SAP B entrained water decreased more quickly compared to the SAP A entrained water signal.
- The amount of mixing water absorbed by the SAP obtained from comparing the flow tests and microscopic analysis is the same as the amount determined by means of NMR.
- The results fit to the model described by Powers and Brownyard^20^. The free water line, however, shows a more concave downward type of curve as a function of the degree of hydration. The results also fit the Feldman and Sereda model^50^ and the Jennings Colloidal Model (CM-II) for C-S-H^52^.
- The main nm pore size distribution was found in the range of 1.5-2 nm followed by an amount of pores with size of 8-12 nm. These can be attributed to the gel pores and the interhydrate pores.
- SAP A is preferable considering the more effective mitigation of self-desiccation and internal curing compared to SAP B. SAP B seems to prematurely release its stored water after time of final setting, while SAP A gradually releases its entrained water in time, thus maintaining the internal relative humidity more effectively.
- The study of the internal relative humidity led to the same conclusions. SAP A is ideal for internal curing and thus the mitigation of autogenous shrinkage.

## References

[1] 225-SAP. Application of Superabsorbent Polymers (SAP) in Concrete Construction. 165 (2012).

[2] Chang C, Duan B, Cai J, Zhang L (2010). Superabsorbent hydrogels based on cellulose for smart swelling and controllable delivery. European Polymer Journal.

[3] Bao Y, Ma J, Li N (2011). Synthesis and swelling behaviors of sodium carboxymethyl cellulose-g-poly(AA-co-AM-co-AMPS)/MMT superabsorbent hydrogel. Carbohydrate Polymers.

[4] Mechtcherine V (2017). Effect of superabsorbent polymers (SAP) on the freeze-thaw resistance of concrete: results of a RILEM interlaboratory test. Mater. Struct..

[5] Mönnig, S. & Lura, P. in *Advances in Construction Materials* (ed C.U. Grosse) Ch. 5, 351–358 (Springer Berlin Heidelberg, 2007).

[6] Mechtcherine V, Secrieru E, Schröfl C (2015). Effect of superabsorbent polymers (SAPs) on rheological properties of fresh cement-based mortars – Development of yield stress and plastic viscosity over time. Cem. Concr. Res.

[7] Lee HXD, Wong HS, Buenfeld NR (2010). Potential of superabsorbent polymer for self-sealing cracks in concrete. Adv. Appl. Ceram..

[8] Lee HXD, Wong HS, Buenfeld NR (2016). Self-sealing of cracks in concrete using superabsorbent polymers. Cem. Concr. Res..

[9] Snoeck D, Steuperaert S, Van Tittelboom K, Dubruel P, De Belie N (2012). Visualization of water penetration in cementitious materials with superabsorbent polymers by means of neutron radiography. Cem. Concr. Res..

[10] Snoeck, D. *Self-Healing and Microstructure of Cementitious Materials with Microfibres and Superabsorbent Polymers* Doctor in Civil Engineering: Construction Design thesis, Ghent University, (2015).

[11] Snoeck D, De Belie N (2015). Repeated autogenous healing in strain-hardening cementitious composites by using superabsorbent polymers. J. Mater. Civ. Eng..

[12] Snoeck D, De Belie N (2015). From straw in bricks to modern use of microfibres in cementitious composites for improved autogenous healing – a review. Constr. Build. Mater..

[13] Snoeck D, Van Tittelboom K, Steuperaert S, Dubruel P, De Belie N (2014). Self-healing cementitious materials by the combination of microfibres and superabsorbent polymers. J. Intel. Mat. Syst. Str..

[14] Jensen OM, Hansen PF (2001). Water-entrained cement-based materials I. Principles and theoretical background. Cem. Concr. Res..

[15] Jensen OM, Hansen PF (2002). Water-entrained cement-based materials II. Experimental observations. Cem. Concr. Res..

[16] Mechtcherine V (2014). Effect of Internal Curing by Using Superabsorbent Polymers (SAP) on Autogenous Shrinkage and Other Properties of a High-performance Fine-grained Concrete: Results of a RILEM Round-robin Test, TC 225-SAP. Materials and Structures.

[17] Snoeck D, Jensen OM, De Belie N (2015). The influence of superabsorbent polymers on the autogenous shrinkage properties of cement pastes with supplementary cementitious materials. Cem. Concr. Res..

[18] Brüdern, A. E. & Mechtcherine, V. in *International RILEM Conference on Use of Superabsorbent Polymers and Other* New *Additives in Concrete*. (eds O.M. Jensen, M.T. Hasholt, & S. Laustsen) 11–22 (RILEM Publications S.A.R.L.).

[19] Bentz, D. P. & Jensen, O. M. Mitigation strategies for autogenous shrinkage cracking. *Cement and Concrete Composites* **26**, 677–685 (2004).

[20] Powers, T. C. & Brownyard, T. L. *Studies of the physical properties of hardened Portland cement paste*. Vol. 22 (Portland Cement Association, Research Laboratories, 1948).

[21] Snoeck D (2015). The effects of superabsorbent polymers on the microstructure of cementitious materials studied by means of sorption experiments. Cem. Concr. Res..

[22] Snoeck D, Schaubroeck D, Dubruel P, De Belie N (2014). Effect of high amounts of superabsorbent polymers and additional water on the workability, microstructure and strength of mortars with a water-to-cement ratio of 0.50. Constr. Build. Mater..

[23] Mönnig S (2005). Water saturated super-absorbent polymers used in high strength concrete. Otto-Graf-Journal.

[24] Trtik, P. *et al*. in *International RILEM Conference on Material Science*. (ed. W. Brameshuber) 175–185 (RILEM Publications S.A.R.L.).

[25] Wyrzykowski M, Lura P, Pesavento F, Gawin D (2011). Modeling of internal curing in maturing mortar. Cem. Concr. Res..

[26] Schröfl C, Mechtcherine V, Vontobel P, Hovind J, Lehmann E (2015). Sorption kinetics of superabsorbent polymers (SAPs) in fresh Portland cement-based pastes visualized and quantified by neutron radiography and correlated to the progress of cement hydration. Cem. Concr. Res..

[27] Pel L, Donkers PAJ, Kopinga K, Noijen JJ (2016). ^1^H, ^23^Na and ^35^Cl imaging in cementitious materials with NMR. Applied Magnetic Resonance.

[28] Muller ACA, Scrivener KL, Gajewicz AM, McDonald PJ (2013). Densification of C-S-H Measured by ^1^H NMR Relaxometry. Physical Chemistry C.

[29] Muller, A. C. A., Scrivener, K. L., Gajewicz, A. M. & McDonald, P. J. Use of bench-top NMR to measure the density, composition and desorption isotherm of C-S-H in cement paste. *Microporous and Mesoporous Materials*, doi:10.1016/j.micromeso.2013.01.032 (2013).

[30] Song Y-Q (2013). Magnetic Resonance of Porous Media (MRPM): A perspective. Journal of Magnetic Resonance.

[31] Brownstein KR, Tarr CE (1979). Importance of classical diffusion in NMR studies of water in biological cells. Physical Review A.

[32] van der Heijden GHA, van Bijnen RMW, Pel L, Huinink HP (2007). Moisture transport in heated concrete, as studied by NMR, and its consequences for fire spalling. Cem. Concr. Res..

[33] Valckenborg RME, Pel L, Kopinga K (2002). Combined NMR cryoporometry and relaxometry. Journal of Physics D: Applied Physics.

[34] Valckenborg RME, Pel L, Hazrati K, Kopinga K, Marchand J (2001). Pore water distribution in mortar during drying as determined by NMR. Mater. Struct..

[35] Hazrati K, Pel L, Marchand J, Kopinga K, Pigeon M (2002). Determination of isothermal unsaturated capillary flow in high performance cement mortars by NMR imaging. Mater. Struct..

[36] Fourmentin M (2017). NMR observation of water transfer between a cement paste and a porous medium. Cem. Concr. Res..

[37] Huang, H., Ye, G. & Pel, L. New insights into autogenous self-healing in cement paste based on nuclear magnetic resonance (NMR) tests. *Mater. Struct*., 1–15, doi:10.1617/s11527-015-0664-9 (2016).

[38] Friedemann K, Stallmach F, Kärger J (2006). NMR diffusion and relaxation studies during cement hydration—A non-destructive approach for clarification of the mechanism of internal post curing of cementitious materials. Cem. Concr. Res..

[39] Nestle N (2009). Water balance and pore structure development in cementitious materials in internal curing with modified superabsorbent polymers studied by NMR. Microporous and Mesoporous Materials.

[40] Schröfl C, Snoeck D, Mechtcherine V (2017). A review of characterisation methods for superabsorbent polymer (SAP) samples to be used in cement-based construction materials - Report of the RILEM TC 260-RSC. Mater. Struct..

[41] Schröfl C, Mechtcherine V, Gorges M (2012). Relation between the molecular structure and the efficiency of superabsorbent polymers (SAP) as concrete admixture to mitigate autogenous shrinkage. Cem. Concr. Res..

[42] Zohuriaan-Mehr MJ, Kabiri K (2008). Superabsorbent Polymer Materials: a Review. Iranian Polymer Journal.

[43] Mechtcherine, V., Dudziak, L. & Hempel, S. in *Creep, Shrinkage and Durability Mechanics of Concrete and Concrete Structures*. (eds R. Sato *et al*.) 847–853 (Taylor & Francis).

[44] Hasholt MT, Jensen OM, Kovler K, Zhutovsky S (2012). Can superabsorbent polymers mitigate autogenous shrinkage of internally cured concrete without compromising the strength?. Constr. Build. Mater..

[45] Laustsen S, Hasholt MT, Jensen OM (2015). Void structure of concrete with superabsorbent polymers and its relation to frost resistance of concrete. Mater. Struct..

[46] Snoeck D, De Belie N (2015). Effect of superabsorbent polymers, superplasticizer and additional water on the setting of cementitious materials. Int. J. of 3R’s.

[47] Song YQ (2002). T(1)–T(2) correlation spectra obtained using a fast two-dimensional Laplace inversion. Journal of Magnetic Resonance.

[48] Parrot LJ, Killoh DC (1984). Prediction of cement hydration. British Ceramic Proceedings.

[49] Jiang Y, Xia Q (2011). Hydration of water entrained cement paste under saturated condition. Applied Mechanics and Materials.

[50] Feldman RF, Sereda PJ (1970). A new model for hydrated portland cement and its practical implications. Engineering Journal.

[51] Jennings HM (2000). A model for the microstructure of calcium silicate hydrate in cement paste. Cem. Concr. Res..

[52] Jennings HM (2008). Refinements to colloid model of C-S-H in cement: CM-II. Cem. Concr. Res..

[53] Trtik P (2011). Release of internal curing water from lightweight aggregates in cement paste investigated by neutron and X-ray tomography. Nucl. Instrum. Meth. A.

[54] Snoeck, D. *et al*. in *14th International Conference on Durability of Building Materials and Components*. 1–8.

